# Biodistribution and Lymphatic Tracking of the Main Neurotoxin of *Micrurus fulvius* Venom by Molecular Imaging

**DOI:** 10.3390/toxins8040085

**Published:** 2016-03-26

**Authors:** Irene Vergara, Erick Y. Castillo, Mario E. Romero-Piña, Itzel Torres-Viquez, Dayanira Paniagua, Leslie V. Boyer, Alejandro Alagón, Luis Alberto Medina

**Affiliations:** 1 Instituto de Biotecnología, Universidad Nacional Autónoma de México, Av. Universidad # 2001, Colonia Chamilpa, Cuernavaca, Morelos 62210, Mexico; ireneveba@gmail.com (I.V.); dashpame@gmail.com (D.P.); alagon@ibt.unam.mx (A.A.); 2 Unidad de Investigación Biomédica en Cáncer INCan-UNAM, Instituto Nacional de Cancerología, Ciudad de México 14080, Mexico; erickcast14@gmail.com (E.Y.C.); esau1708@gmail.com (M.E.R.-P.); itzel2606@gmail.com (I.T.-V.); 3 Venom Immunochemistry, Pharmacology and Emergency Response (VIPER) Institute, University of Arizona College of Medicine, Tucson, AZ 85724, USA; boyer@viper.arizona.edu; 4 Instituto de Física, Universidad Nacional Autónoma de México, Ciudad de México 04510, Mexico

**Keywords:** coral snake, neurotoxin, lymphatic absorption, molecular imaging, radiolabeling

## Abstract

The venom of the Eastern coral snake *Micrurus fulvius* can cause respiratory paralysis in the bitten patient, which is attributable to β-neurotoxins (β-NTx). The aim of this work was to study the biodistribution and lymphatic tracking by molecular imaging of the main β-NTx of *M. fulvius* venom. β-NTx was bioconjugated with the chelator diethylenetriaminepenta-acetic acid (DTPA) and radiolabeled with the radionuclide Gallium-67. Radiolabeling efficiency was 60%–78%; radiochemical purity ≥92%; and stability at 48 h ≥ 85%. The median lethal dose (LD_50_) and PLA_2_ activity of bioconjugated β-NTx decreased 3 and 2.5 times, respectively, in comparison with native β-NTx. The immune recognition by polyclonal antibodies decreased 10 times. Biodistribution of β-NTx-DTPA-^67^Ga in rats showed increased uptake in popliteal, lumbar nodes and kidneys that was not observed with ^67^Ga-free. Accumulation in organs at 24 h was less than 1%, except for kidneys, where the average was 3.7%. The inoculation site works as a depot, since 10% of the initial dose of β-NTx-DTPA-^67^Ga remains there for up to 48 h. This work clearly demonstrates the lymphatic system participation in the biodistribution of β-NTx-DTPA-^67^Ga. Our approach could be applied to analyze the role of the lymphatic system in snakebite for a better understanding of envenoming.

## 1. Introduction

The *Micrurus* genus belongs to the Elapidae family and it includes nearly 60 different coral snake species native to the Americas. *Micrurus fulvius* is endemic to the United States. It possesses neurotoxic venom, which can lead to respiratory paralysis in severe envenoming [1,2]. Signs and symptoms in humans include: local pain, sialorrhea, paresthesia, ptosis, weakness, blurred vision, paralysis, fasciculation, and diplopia [2,3]. In contrast with other coral snake species, *M. fulvius* venom contains only small amounts of Three Finger Toxins (3FTx), which are not lethal to mice; its mammalian neurotoxicity is instead attributable to high amounts of lethal β-neurotoxins (β-NTx) with phospholipase A_2_ (PLA_2_) activity [4]. The most lethal (LD_50_ = 0.54 µg/g) and abundant component (18% of venom content) is a β-NTx of 13,436 Da [4].

Analyses of biodistribution and kinetics are essential to understand the envenomation process and to improve diagnosis and treatment. Typically, in experimental envenomation studies, the venom or isolated toxins are injected intravenously (IV). However, the IV route does not mimic a real envenomation, since snakes usually inject venom into subcutaneous or intramuscular sites. After SC injection, proteins are readily absorbed through blood or lymphatic vessels, with the proportion essentially determined by molecular weight: as molecular weight rises, lymphatic absorption dominates [5,6,7]. Although lymphatic absorption of therapeutic proteins has been extensively analyzed, there exist extremely few studies of lymphatic participation in the absorption of animal venoms.

Our group reported the pharmacokinetics (PK) of *M. fulvius* venom in anesthetized sheep [8]. In this study, whole venom was administered subcutaneously, after which blood and lymph samples were collected over the course of six hours. The PK analysis revealed incomplete recovery of the injected venom, with 60% accounted for in blood and lymph, and 3% in organs and urine. The residual venom (*i.e*., nearly of 30% of the injected dose) is believed to have remained at the injection site [8]. Unfortunately, measurement of venom kinetics in anesthetized sheep cannot be continued beyond 6–8 h, due to the physiological limitations of the animal model; *i.e.*, it is difficult to maintain stable anesthesia in a ruminant animal, because of gas accumulation in the stomach and intestines. Furthermore, venom metabolism and elimination could not be fully evaluated because the method of quantification (immunoassay ELISA) does not detect metabolized venom. Limitations of the sheep model encouraged our research group to explore different strategies to study the biodistribution and lymphatic absorption of *M. fulvius* venom over a longer period of time.

Pre-clinical molecular imaging techniques using the hybrid system SPECT/CT (Single Photon Emission Computed Tomography/Computed Tomography) are useful to study the anatomic distribution of radiolabeled biomolecules *in vivo*, in real time [9]. This technology has established applicability to lymphatic diseases. Therefore, the SPECT/CT technique could provide valuable information in the field of the toxinology, especially regarding the role of the lymphatic system in snakebites.

The aim of this work was to standardize methods for bioconjugation and radiolabeling of β-NTx from *M. fulvius* venom, to study the biodistribution and to analyze the role of the lymphatic system in envenomation, by molecular imaging. This approach would allow better understanding of envenomation progression and will be precedent to study other venom toxins.

## 2. Results

### 2.1. Isoelectric Point (pI) of β-NTx

**Figure 1:**
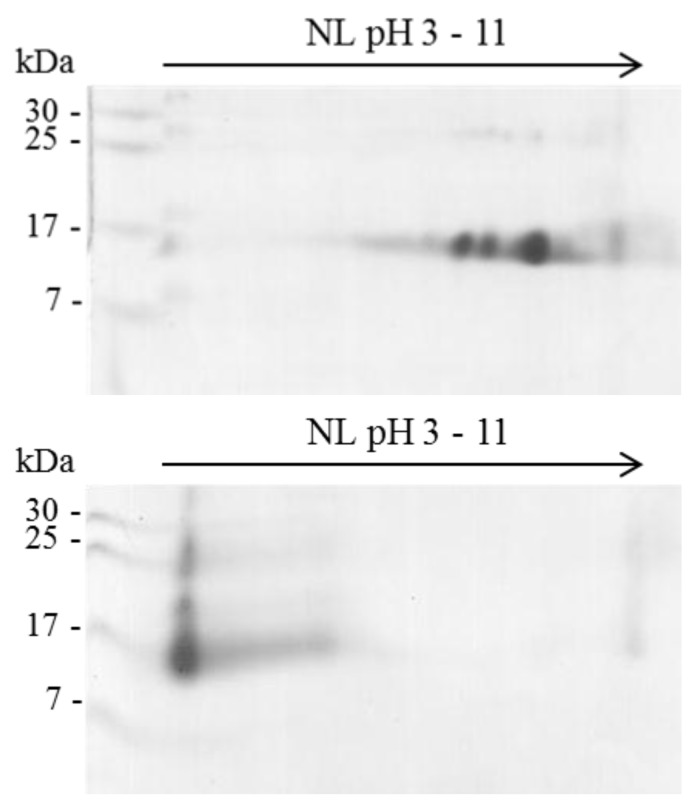
Electrophoretic profile 2D-PAGE of native β-NTx (**Top**) and β-NTx-DTPA (**Bottom**), IEF using a wide pH range (3–11 Non Linear-NL-IPG strip) and 17% SDS-PAGE for the second dimension.

Two-dimensional-gels were used to determine the effect on isoelectric point of the β-NTx as result of the bioconjugation process. Native β-NTx has several isoforms at basic pH (8–10) (Figure 1 (Top)), while functionalized β-NTx is mainly located at pH 3 due to carboxyl groups in the DTPA bound (Figure 1 (bottom)), as it was observed in the 2D-PAGE gel. It also shows the formation of complexes with molecular weight higher than native β-NTx, probably as result of cross-linked process from several molecules of β-NTx and DTPA, as has been reported for functionalized biomolecules with DTPA [10,11].

### 2.2. Conjugation Efficiency

Atomic absorption analysis showed that on average there were 2.5 atoms of Cu^2+^ for each molecule of β-NTx-DTPA. This implied that at least 2.5 chelating agents (DTPA) were linked to each neurotoxin. For DTPA-BSA, results showed five atoms of Cu^2+^ bound to each molecule of albumin. 

### 2.3. Changes in Biological Activities of B-NTx

The lethality of β-NTx-DTPA decreased to one third, relative to that of the native toxin. Enzymatic activity was 675 and 278 U/mg for native and functionalized β-neurotoxin, respectively. This means that catalytic activity decreased 2.4 fold; perhaps related to cross-linked complexes that might be hindering catalytic action.

### 2.4. Immuno-Recognition of β-NTx-DTPA

**Figure 2:**
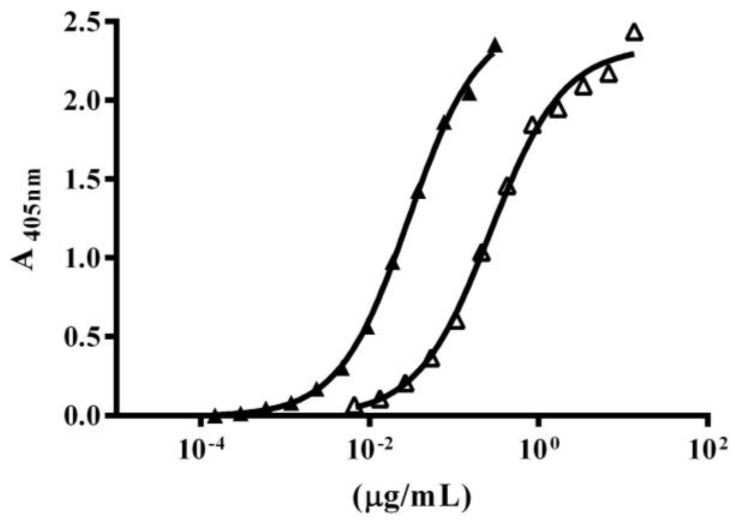
ELISA-sandwich dose-response curve. Black triangles represent Native β-NTx, Open triangles represent β-NTx-DTPA.

ELISA assay shows high recognition of native β-NTx by polyclonal antibodies against *M. fulvius* venom with EC_50_ 0.0289, while recognition of the β-NTx-DTPA decreased ten times (EC_50_ = 0.268) (Figure 2). A decrease in immune-recognition could be related to the steric effect caused by DTPA, which might disrupt the binding of the antibodies to available epitopes.

### 2.5. Radiolabeling Efficiency and Stability

Radiolabelling efficiency obtained in several experiments was 60%–78%, radiochemical purity was ≥92%, and stability in human serum at 48 h was ≥85%. The stability in human serum is consistent with data previously reported, which indicates 85% of stability in human plasma during 50 h for an antibody functionalized with DTPA and radiolabeled with Indium-111 (^111^In) [12]. Our results demonstrate that radiolabeled bioconjugate is acceptable and reliable for *in vivo* assays.

### 2.6. β-NTx-DTPA-^67^Ga Disappearance from Injection Site

**Figure 3:**
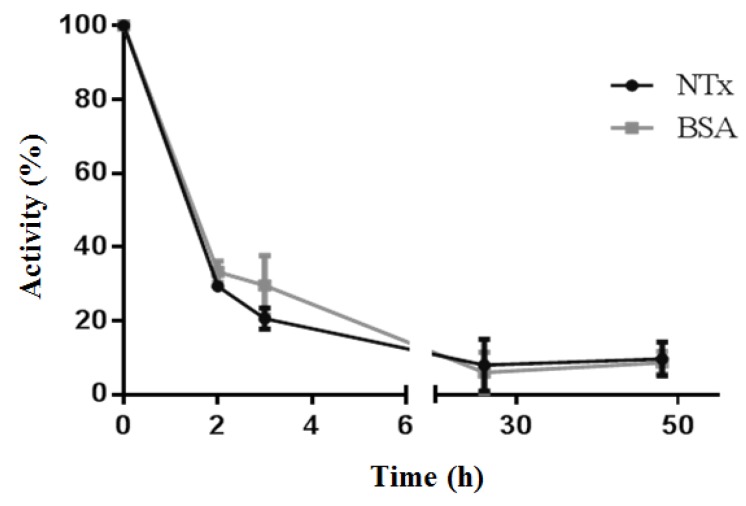
Remaining activity at the injection site in rats of the β-NTx-DTPA-^67^Ga and BSA-DTPA-^67^Ga.

Three hours post-injection of β-NTx-DTPA-^67^Ga, the remaining activity at the injection site fell to 20% of initial activity, and 48 h later it was approximately 10% (Figure 3). This result is noteworthy because residual venom at the site could continue to be absorbed into the bloodstream for an extended time. This finding supports the hypothesis that the injection site works as a reservoir for extended release of the toxin.

### 2.7. β-NTx-DTPA-^67^Ga Biodistribution by Molecular Imaging

**Figure 4:**
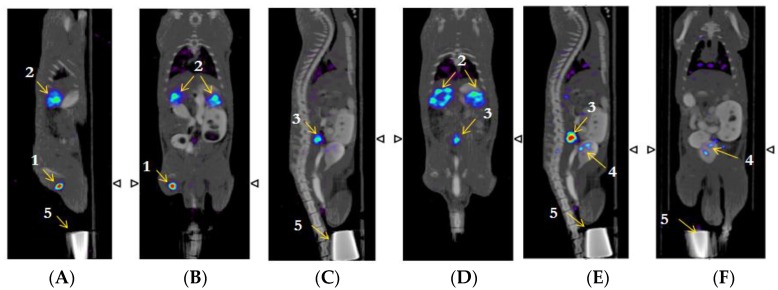
Biodistribution of β-NTx-DTPA-^67^Ga by molecular image (SPECT/CT) 3 h post-injection; image representative of four individual experiments. (**A**) and (**B**) are sagittal and coronal slices, respectively, showing uptake in the popliteal node (labeled as 1) and kidneys (labeled as 2); (**C**) is a sagittal slice demonstrating uptake in the lumbar node (labeled as 3); (**D**) is a different sagittal slice, showing accumulation in lumbar node and kidneys; (**E**) demonstrates accumulation in lumbar node and bladder (labeld as 4); (**F**) shows the accumulation in bladder. Number 5 shows the injection site, masked by a lead shield.

**Figure 5:**
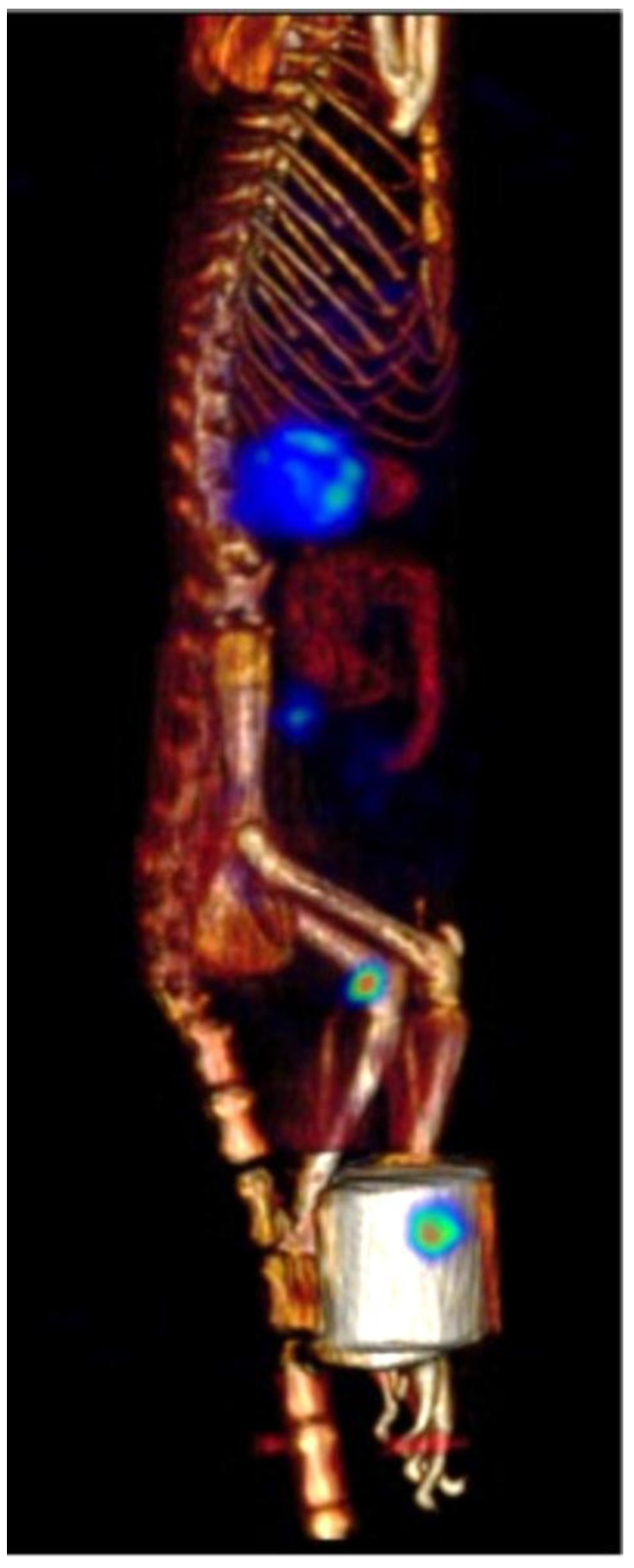
SPECT/CT 3D-imaging depicting biodistribution of β-NTx-DTPA-^67^Ga in the rat described in Figure 4.

Sagittal and coronal images showed that 3 h following injection of β-NTx-DTPA-^67^Ga (Figure 4), there is an increased uptake in popliteal and lumbar nodes (labeled as 1 and 3, respectively) and kidneys (labeled as 2), which is consistent with the maximum accumulation found in dissected tissue after sacrifice of rats. Figure 5 depicts a 3D image of the same rat.

**Figure 6:**
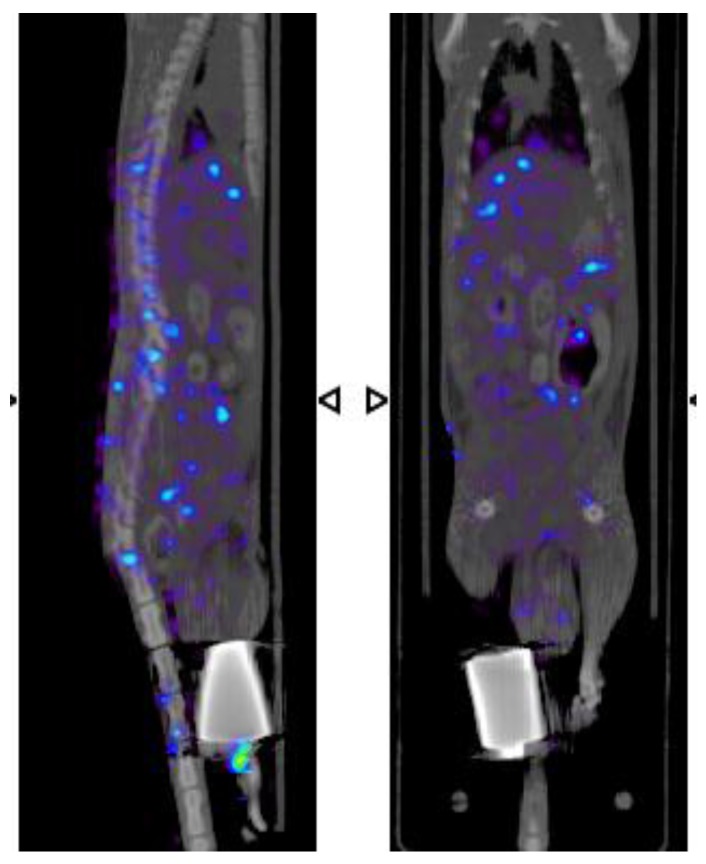
Sagittal and coronal images, respectively, demonstrate the biodistribution of ^67^Ga-free 3 h post-injection.

After 24 h, accumulation in popliteal node and kidneys decreased, and most of the injected dose had already been eliminated by urine. These results clearly confirm the participation of the lymphatic system in the absorption and distribution of venom, since ^67^Ga-free (Figure 6) showed a broad distribution in blood circulation. Table 1 shows the percentage of β-NTx-DTPA-^67^Ga, BSA-DTPA-^67^Ga and ^67^Ga-free (the last two used as controls) in terms of the injected dose per gram of tissue in organs, urine and feces 24 h after injection.

## 3. Discussion

In past decades, PK studies of animal toxins radiolabeled with Iodine-125 (^125^I) or Technetium-99m (^99m^Tc) have yielded important findings in the toxinology field [13,14,15]. For our purpose, the physical properties of those radionuclides imposed some limitations on the performance of studies with imaging techniques or PK studies longer than 24 h. For instance, ^125^I has a long half-life (59.4 days) but its gamma energy (35.4 keV) does not work with common SPECT (Single Photon Emission Computed Tomography), while ^99m^Tc possesses excellent gamma energy (140 keV) but has a short half-life (6 h). In contrast, ^67^Ga has physical properties that are desirable and suitable to PK and biodistribution studies using molecular imaging, including half-life (3.2 days) and three principal gamma rays (93, 184 and 300 keV). This work shows, for the first time, the use of ^67^Ga as a promising radionuclide to label other toxins from venoms or, potentially, antivenoms for biodistribution and PK studies. 

β-NTx and BSA were bioconjugated with DTPA by deprotonation of the ε-amino of lysine to form an amide bond by nucleophilic attack to one carboxyl of the activated DTPA. The reaction produced 2.5 and 5 DTPAs per β-NTx and BSA, respectively. Higher amounts of DTPAs found in BSA could be explained by the number of Lys contained in the primary structure of protein; since β-NTx contains eleven lysines [16] while BSA has 58 [17], which increase the probability of binding to DTPAs. 

Changes in LD_50_ and enzymatic activity might result from the formation of high molecular weight complexes observed in 2D-gel (Figure 1) and from the chemical modification of important amino acid residues involved in toxicity or enzyme activity. Although PLA_2_ activity is necessary to trigger the neurotoxic effect [18], no proportional correlation has been found, since some toxins with low PLA_2_ activity are highly lethal [19]. In this case, however, the lethal effect in mice decreased proportionally to PLA_2_ activity. Some authors propose to assess different molar proportions of peptide: chelate or add some “linker” between the biomolecule and chelate to preserve the biological activity [20,21]. Use of “linkers” could be considered in future studies to understand the effect of bioconjugation process on enzymatic and lethal activity of β-NTx. 

Immunoreactivity among polyclonal antibodies and β-NTx-DTPA decreased 10 times as compared to native β-NTx, as EC_50_ showed. This result might be due to masking epitopes by DTPA, plus some DTPA-β-NTx aggregates. More thorough experiments are needed to clarify this point.

The residual β-NTx-DTPA-^67^Ga at the injection site confirmed that the site can serve as a reservoir for extended-release, known in the field of venoms as “venom depot” [22]. This phenomenon has already been observed in snakebite caused by other elapid species [23]. There have been clinical reports of envenomation in which antivenom was administered and patients, apparently recovered, were discharged to home. Some hours later, patients returned to the hospital presenting with recurrent signs of envenomation. Medical toxinologists have suggested that recurrence of envenomation is caused by active toxins, retained at the bite site, which are slowly delivered into the bloodstream [22,24,25]. Our results support that conclusion. 

Biodistribution of biomolecules in organs or tissues is influenced by size, target specificity and affinity, solubility, route of administration, and other factors [26,27]. In this work, the greatest accumulation in popliteal and lumbar nodes was observed 3 h after injection (Figure 4), clearly demonstrating the role of the lymphatic system in the absorption of β-NTx-DTPA-^67^Ga injected subcutaneously, while the control group (^67^Ga-free) showed biodistribution mainly in blood, as expected (Figure 6). On the other hand, the accumulation of β-NTx in kidneys and urine was unexpected, given our previous detection of only 0.12%–0.22% of *M. fulvius* venom in urine, by ELISA, in sheep [8]. It may be that a significant amount of β-NTx is catabolized in the kidneys and that the resulting digestion products are rendered undetectable by the assay or that bioconjugated β-NTx has a different renal secretion pattern as compared to the native one.

Our understanding of the role of the lymphatic system in snakebites is still poor; however, recent research has provided some further insights. In 2008, the effect of *Bothrops asper* venom on the structure and function of mouse mesenteric collecting lymphatic vessels was assessed. The conclusion was that *B. asper* venom induced a rapid reduction in the lumen of these vessels; and the effect was reproduced by a myotoxic phospholipase A_2_ isolated from this venom [28]. In 2012, the absorption of *M. fulvius* venom through the lymphatic route was quantified in a sheep model [8], and, in 2014, a study investigated the inhibition of lymphatic flow using drugs as adjunct treatment to pressure bandaging with immobilization in snakebite first aid [29]. All these findings could have important implications for the management of snakebite envenomation, particularly in elapids; because we know that the lymphatic system is a key route for absorption and biodistribution of snake venoms. However, important questions remain to be answered, including: what are the implications of lymphatic absorption on the PK of venoms? What effects do different snake venom components have on lymphatic system function? Additionally, does the reduction of lymphatic flow in the first aid management of elapid (neurotoxic) envenomations provide actual clinical benefit?

## 4. Experimental Section

### 4.1. Reagents and Venom 

The bifunctional chelator DTPA, Streptavidin Peroxidase (from *Streptomyces avidinii*), Bovine Serum albumin (BSA) and all buffers were purchased from Sigma-Aldrich Co. (St. Louis, MO, USA). Chelex 100 resin was purchased from Bio-Rad Laboratories Inc. (Hercules, CA, USA). The 2,2’-azino-bis(3-ethylbenzothiazoline-6-sulphonic acid) (ABTS) was from Research Organics Inc. (St, Cleveland, OH, USA). The polyclonal antibodies for the ELISA immunoassay were produced in our laboratory by hyperimmunization of rabbits; these antibodies were then immunopurified by affinity chromatography and a portion of them biotinated using EZ-link NHS-LC-Biotin from Thermo Fisher Scientific Inc. (Waltham, MA, USA) [8]. Pure water (18 MΩ cm^−1^), passed through a column containing Chelex 100 to remove trace metals, was used to prepare all buffers. The most abundant and lethal β-neurotoxin of *Micrurus fulvius* venom was isolated and characterized, as previously reported [4], from a venom pool purchased from the National Natural Toxins Research Center (NNTRC, Texas A&M University-Kingsville, Kingsville, TX, USA). Radionuclide (^67^GaCl_3_) was purchased from a local radiopharmacy Medidores Industriales y Médicos S.A. de C.V. (MYMSA, Ciudad de Mexico, Mexico).

### 4.2. Animals 

Male Wistar rats (250–300 g) and CD1 mice (18–20 g) were purchased from Harlan Mexico. Animals received water and food *ad libitum* and were maintained under a 12-h light/dark cycle. The local Institutional Committee for Animal Welfare approved the experimental protocol for animal handling, identification code: INCAN/CB/013/10) (CB/619), approved at 29 April 2010.

### 4.3. Bioconjugation of β-NTx or BSA with DTPA Chelator 

Bioconjugation was carried out according to a well-established method [30] with slight modifications. Briefly, 3 mg of neurotoxin or 5 mg of BSA were conjugated with DTPA anhydride and dissolved in DMSO at molar ratio 1:10. The reaction was performed in 0.1 M sodium bicarbonate pH 8.3 and incubated 1 h at room temperature. Then, the mixture was exhaustively dialyzed with 0.1 M NaOAc pH 7.5, followed by dialysis against 0.25 M NH_4_OAc pH 4.7. Bioconjugated proteins were stored at −20 °C until use.

### 4.4. Bidimensional Electrophoresis (2D-PAGE)

The isoelectric point (pI) of β-neurotoxin was determined by 2D electrophoresis before and after bioconjugation as previously reported [4]. Briefly, the Isoelectric Focusing (IEF) (first dimension) was performed at 20 °C in 13 cm Immobiline Dry Strips with non-linear pH gradient from 3 to 11. The IEF protocol was developed under voltage gradient with current limit of 75 µA per strip. Voltage was linearly increased from 1000 to 8000 V by 2.5 h for sample entry, followed by a constant 8000 V and sample focused complete after 15,000 V/h. The second dimension was performed on bis-acrlyamide gel (17%). 

### 4.5. Atomic Absorption Spectroscopy 

Atomic absorption spectroscopy was used as indirect method to quantify the DTPA attached to BSA or β-NTx. For this purpose, a complex of Cu^2+^-DTPA-protein was performed with 200 µg of bioconjugated protein in AcONH_4_ 0.25 M pH 4.7 plus CuSO_4_ ·H_2_O in molar rate 1:10. Reaction was incubated 1 h at 37 °C and then exhaustively dialyzed against AcONa 0.1 M pH 6.7. Controls were native proteins without bioconjugation submitted to the same procedure. Copper (Cu^2+^) amount was determined by a certified laboratory in chemical analysis (Unidad de Servicios de Apoyo a la Investigación) from the Universidad Nacional Autónoma de México (Ciudad de Mexico, Mexico). Bioconjugation-efficiency was calculated assuming that copper atoms found in complex Cu^2+^-DTPA-protein, minus those quantified in the control (native proteins), are proportional to DTPAs attached.

### 4.6. Biological Assays

The medium lethal dose (LD_50_), either native or bioconjugated β-neurotoxin, was determined in groups of five CD1 mice after intravenous (IV) injection in the tail. The control group was injected with NaCl 150 mM as vehicle solution. Mortality was recorded at 48 h post-injection. The LD_50_ value was calculated by sigmoidal dose-response curve with non-linear regression, using the Grad Pad Prism (Version 4.0, GraphPad Software Inc., San Diego, CA, USA, 1994–2003). 

The enzymatic activity PLA_2_ of the β-neurotoxin was measured before and after bioconjugation by a titrimetric method previously reported [4]. Briefly, samples were evaluated using 10% egg yolk solution (NaCl 0.1 M, CaCl_2_ 0.01 M, 0.1% Triton X-100) as substrate and titred with 50 mM NaOH. Specific activity was reported in units defined as μmoles of NaOH consumed per minute per milligram of protein (U/mg). 

An ELISA sandwich immunoassay was performed to compare the immune-recognition of native or bioconjugated β-NTx by polyclonal antibodies against *M. fulvius* venom. The ELISA assay was performed in MaxiSorp™ 96 well plates (Nalgene NUNC™, Rochester, NY, USA), as previously described [8]. Samples were diluted in immunoassay vehicle solution (100 mM NaCl, 0.1% gelatin, 0.05% Tween 20, in 50 mM Tris/HCl buffer at pH 8) and serial dilutions (1:2) were performed. A standard curve of native β-NTx was carried out starting with 300 ng/mL and followed by serial dilutions 1:2.

### 4.7. Radiolabeling

Radiolabeling was achieved by reaction of the bioconjugated protein (200–250 µg) plus 1 mCi of ^67^GaCl_3_ at pH 5.5 (adjusted with 1 M AcONH_4_ pH 6.7) followed by incubation 1 h at 37 °C. The radiolabeled complex was purified and the buffer was exchanged to PBS using Amicon ultracel filter (10 kDa) from Millipore (Darmstadt, Germany) [31]. 

Radiochemical purity was assessed by instant Thin Layer Chromatography (iTLC-SG, Agilent Technologies, Santa Clara, CA, USA). A sample of radiolabeled complex (2 µL) was spotted on the chromatography sheet paper, using AcONH_4_ 0.25 M pH 4.7 with DTPA (1µM) as developing solvent. The strip was cut by half and each segment quantified in a well counter scintillation (Ludlum Measurements Inc., Sweetwater, TX, USA). Independent iTLC of ^67^GaCl_3_ was run as a control. Under conditions described above, the bioconjugate remains at the origin, while ^67^Ga-free migrate with solvent front. Radiochemical stability was determined after incubation of radiolabeled bioconjugate in PBS or fresh human serum (1 mL) at 37 °C during 48 h. Dissociation of ^67^Ga was evaluated at different times by iTLC as described above.

### 4.8. Imaging Studies

A microPET/SPECT/CT system (Albira ARS, Oncovision, Valencia, Spain) was employed to analyze the biodistribution of β-NTx-DTPA-^67^Ga in normal rats (*n* = 4). The radiolabeled neurotoxin was injected subcutaneously into the hind paw of rats under anesthesia with isofluorane 3%. Each rat received 100 µg of neurotoxin containing 360 (±50) µCi of ^67^Ga. To compare and differentiate the biodistribution of the radionuclide (^67^Ga-free), a second group of rats was injected with 230 (±15) µCi of pH 7-neutralized ^67^GaCl_3_. A double energy window, centered at 93 and 184 keV, was used for SPECT imaging acquisition. Home-made shielding with Cerrobend alloy was added to the collimator-detectors to reduce artifacts produced by scatter radiation from the high energy gammas from the ^67^Ga. Planar whole-body images (128 × 128 matrix) were acquired at 0, 1, 3, 5, and 24 h post-injection; tomographic SPECT/CT images were performed at 3 h (60 projection/30 s). To improve identification of abdominal soft-tissue organs in CT image, twelve hours before imaging, animals were fed with food chips impregnated with iodined contrast (Optiray™ 320, Mallinckrodt Pharmaceuticals, Dublin, Ireland). 

Quantification of the remaining dose at injection site was evaluated in another experiment; planar images of the rat-paw (*n* = 3 rats) were taken at different times (0, 1.5, 3, 5, 26 and 48 h). As a control to compare neurotoxin movement and retention at the injection site, we used BSA protein since it is commonly used as a colloid to assess lymphatic tracking. For this purpose, β-NTx-DTPA-^67^Ga complex was subcutaneously injected in the right paw and BSA-DTPA-^67^Ga in the left paw. The amount of either β-NTx-DTPA-^67^Ga or BSA-DTPA-^67^Ga remaining at the injection site, at each specific time point, was determined by quantifying the radioactivity at region of interest (ROI) and considering ^67^Ga decay correction. 

### 4.9. Biodistribution 

The biodistribution of β-NTx-DTPA-^67^Ga, BSA-DTPA-^67^Ga or ^67^Ga-free was performed in groups of four rats, respectively. Each rat received approximately 100 µg of neurotoxin (360 ± 50 µCi), 100 µg of BSA (405 ± 55 µCi) or neutralized ^67^Ga (230 ± 15 µCi). Rats were housed in metabolic cages for 24 h with water and food, and then sacrificed by neck dislocation. Major organs were removed and total feces and urine collected for quantification of radioactivity. The radioactivity was measured in a shielded well-type gamma counter and corrected by gamma decay. Biodistribution was expressed as percent of total injected dose.

## 5. Conclusions

Bioconjugation and radiolabeling of the main β-NTx of *M. fulvius* venom was implemented and standardized to biodistribution studies by molecular image. Our results suggest that the venom injection site works as a venom depot, releasing the β-NTx for at least 48 h with participation of the lymphatic system in the absorption process. The results also suggest that metabolism and elimination of the neurotoxin may be kidney dependent; however, more experiments are necessary to confirm this hypothesis. This work has shown that molecular imaging using SPECT/CT allows tracking the radiolabeled β-NTx *in vivo* and at real time for at least 48 h. Therefore, this approach could be applied to study other toxins or venoms and could contribute to better understanding of envenomation

## Figures and Tables

**Table 1:** Percentage of injected dose accumulated in organs, urine and feces 24 h post-injection.

Organ	β-NTx-DTPA-^67^Ga	BSA-DTPA-^67^Ga	^67^Ga
	% Dose/g Tissue	% Dose/g Tissue	% Dose/g Tissue
Kidneys	3.15 (±0.77)	1.96 (±0.69)	1.82 (±0.19)
Heart	0.13 (±0.03)	0.08 (±0.02)	0.04 (±0.01)
Lung	0.47 (±0.20)	0.14 (±0.06)	0.04 (±0.01)
Liver	1.42 (±0.75)	0.64 (±0.02)	0.17 (±0.03)
Diaphragm	0.10 (±0.04)	0.04 (±0.01)	0.03 (±0.01)
Stomach	0.21 (±0.10)	0.16 (±0.07)	0.04 (±0.01)
Spleen	0.76 (±0.30)	0.60 (±0.11)	0.32 (±0.19)
**Sample**	**% Dose**	**% Dose**	**% Dose**
Urine	32.1 (±3.0)	33.7 (±12.0)	61.3 (±4.5)
Feces	2.23 (±0.66)	3.1 (±1.6)	5.47 (±2.0)
